# *Lycopersicon esculentum* Extract Enhances Cognitive Function and Hippocampal Neurogenesis in Aged Mice

**DOI:** 10.3390/nu8110679

**Published:** 2016-10-26

**Authors:** Jung-Soo Bae, Mira Han, Hee Soon Shin, Dong-Hwa Shon, Soon-Tae Lee, Chang-Yup Shin, Yuri Lee, Dong Hun Lee, Jin Ho Chung

**Affiliations:** 1 Department of Dermatology, Seoul National University College of Medicine, 101, Daehak-ro Jongno-gu, Seoul 03080, Korea; ralral82@naver.com (J.-S.B.); mira0221@nate.com (M.H.); shiny69@empal.com (C.-Y.S.); lepommier@snu.ac.kr (Y.L.); 2 Laboratory of Cutaneous Aging Research, Biomedical Research Institute, Seoul National University Hospital, 101, Daehak-ro Jongno-gu, Seoul 03080, Korea; 3 Institute of Human-Environment Interface Biology, Seoul National University, 101, Daehak-ro Jongno-gu, Seoul 03080, Korea; 4 Korea Food Research Institute, Seongnam-si, Kyeonggi-do 13539, Korea; hsshin@kfri.re.kr (H.S.S.); dhs95@kfri.re.kr (D.-H.S.); 5 Food Biotechnology Program, Korea University of Science and Technology, Daejeon 34113, Korea; 6 Department of Neurology, Seoul National University College of Medicine, 101, Daehak-ro Jongno-gu, Seoul 03080, Korea; staelee@gmail.com; 7 SNU Institute on Aging, Seoul National University, 101, Daehak-ro Jongno-gu, Seoul 03080, Korea

**Keywords:** aging, brain-derived neurotrophic factor, hippocampus, memory function, neurogenesis, tomato ethanolic extracts

## Abstract

A decrease in adult neurogenesis is associated with the aging process, and this decrease is closely related to memory impairment. Tomato (*Lycopersicon esculentum*) is a fruit with diverse bioactive nutrients that is consumed worldwide. In this study, we investigated the cognition-enhancing effect of tomato ethanolic extracts (TEE) in aged mice. Six weeks of oral TEE administration in 12-month-old aged mice significantly increased their exploration time of novel objects when compared to vehicle-treated mice. The TEE supplement increased doublecortin (DCX)-positive cells and postsynaptic density-95 (PSD95) expression in mice hippocampus. Moreover, we found an increased expression of brain-derived neurotrophic factor (BDNF) and subsequently-activated extracellular-signal-regulated kinase (ERK)/cAMP response element binding (CREB) signaling pathway in the TEE-supplemented mice hippocampus. In conclusion, the oral administration of TEE exhibits a cognition-enhancing effect, and the putative underlying mechanism is the induction of BDNF signaling-mediated proliferation and synapse formation in the hippocampus. These findings indicate that TEE could be a candidate for treatment of age-related memory impairment and neurodegenerative disorders.

## 1. Introduction

Neurogenesis is a process that consists of the generation of neurons from neural stem cells and progenitor cells. The subgranular zone (SGZ) in the hippocampus is a major part of the brain involved in adult neurogenesis [1]. Neurogenesis is most active during pre-natal development, and it declines substantially with age [2]. As such, neurodegeneration is one of the most common features of aging, and numerous aged individuals suffer from cognitive deficits such as memory impairment, Alzheimer’s disease and depression [3]. The hippocampus plays an important role in learning and memory, and its function and histologic integrity are known to deteriorate with age [4]. There is considerable histologic evidence of relationships among aging, hippocampal atrophy, and synaptic degeneration [5], indicating that age-related hippocampal deterioration can be considered as a cause of memory decline.

Previous studies have demonstrated the neurogenic and cognition-enhancing activities of several plant-derived agents [6,7]. The brain is influenced by several hormones and growth factors that affect the generation, maturation and survival of neurons [8]. Many neurotrophic factors decline with age, and some agents increase cognitive function by up-regulating neurotrophic factors, such as the nerve growth factor (NGF) and brain-derived neurotrophic factor (BDNF) [9,10]. BDNF is a neurotrophic factor that regulates not only the growth and differentiation of newborn neurons, but also the synaptic plasticity via activation of signaling pathways involved in the release of neurotransmitters [11]. In addition, BDNF activates extracellular-signal-regulated kinase 1/2 (ERK1/2)/cAMP response element binding (CREB) signaling pathway that is involved in hippocampal neurogenesis and memory formation [12]. Therefore, BDNF has been a potent therapeutic target to improve memory.

Tomato (*Lycopersicon esculentum*) is consumed worldwide as a raw fruit or in processed products, and it is an important dietary source of nutrients such as polyphenols, flavonoids and several antioxidants. Diverse active compounds in tomato have shown pharmacological efficacy in several types of cancer [13], endothelial function [14], obesity [15] and neuroprotection [16]. Recent reports have demonstrated that tomato seed extract reduced oxidative stress and neurotoxicity in a rotenone-induced Parkinson’s disease (PD)-like mouse model [17]. Lycopene (a major antioxidant in tomato) also has a neuroprotective effect on 1-methyl-4-phenyl-1,2,3,6-tetrahydropyridine (MPTP) -induced PD in mice through improvement of mitochondrial impairment and inflammation [18]. However, the neurogenic and cognitive-enhancing effects of tomato in aged mice has not yet been elucidated.

Here, we investigate the effects that tomato has on the cognitive function of 12-month-old aged mice as well as the underlying molecular mechanism. To obtain a polar and non-polar substance, tomato was extracted with 50% ethanol and the extracts were then used. We performed a novel object recognition (NOR) test after administration of tomato ethanolic extracts (TEE) for six weeks and investigated the expression of BDNF and the relevant signaling pathway in the hippocampus.

## 2. Materials and Methods

### 2.1. Antibodies

The antibodies used in this study were as follows; mouse monoclonal antibodies against postsynaptic density-95 (PSD-95; ab2723, Abcam, Cambridge, UK), goat polyclonal antibodies against β-actin (sc-1616, Santa Cruz Biotechnology, Santa Cruz, CA, USA), goat polyclonal antibodies against doublecortin (DCX; sc-8066, Santa Cruz Biotechnology), rabbit polyclonal antibodies against phospho-ERK (#9101S, Cell Signaling Technology, Danvers, MA, USA) and polyclonal antibodies against total ERK (#9102, Cell Signaling Technology).

### 2.2. Preparation of Tomato Ethanolic Extract (TEE)

TEE was prepared by the Korea Food Research Institute (Seongnam-si, Korea). Tomatoes were sliced, shade dried, powdered, and sequentially extracted with 50% alcohol twice for 3 h each. The extract was filtered, condensed by a vacuum, and freeze-dried at −40 °C under a reduced pressure (yield: 46.18% of dry weight).

### 2.3. Animals and TEE Administration

**Figure 1:**
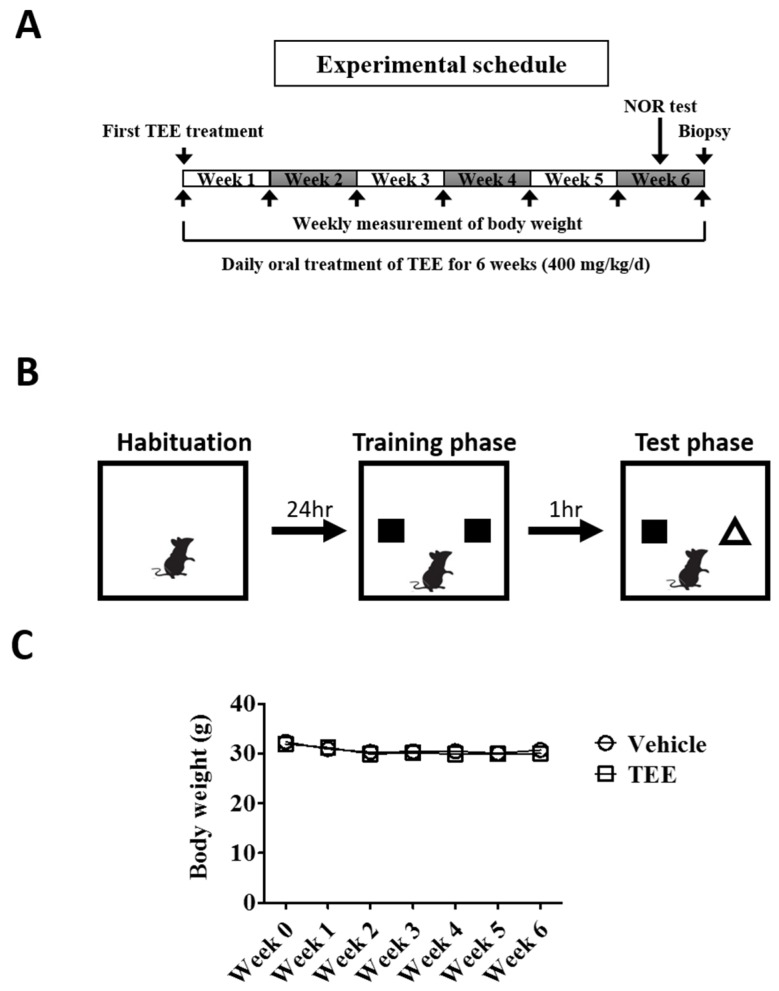
Study design and body weight changes of mice during the study. (**A**) Experimental schedule. Vehicle or tomato ethanolic extract (TEE) was orally administered to 12-month-old aged mice once a day for 6 weeks. Two days before biopsy, the mice were subject to a novel object recognition (NOR) test; (**B**) Schematic representation of the NOR test protocol. On the first day, each mouse was placed in a test chamber for 5 min (habituation phase). The next day, the mouse was allowed to explore two identical objects for 5 min (training phase), followed by a 1 h interval, and a subsequent test phase with one familiar and one novel object for 5 min (testing phase); (**C**) Changes in body weight of the mice during the study. Each point represents the mean ± standard error of the mean (SEM) for each group (*n* = 9).

Twelve-month-old and eight-week-old young female albino hairless mice (Skh-1) were purchased from Orient Bio (Seongnam-si, Korea). The animals were allowed to feed *ad libitum* and were acclimated for one week prior to the study. All experimental protocols were approved by the Institutional Animal Care and Use Committee of the Center for Phenogenomics Animal Research, Woojung BSC (Suwon, Korea, accredited by the Association for Assessment and Accreditation of Laboratory Animal Care). The aged mice were randomly divided into two equivalent groups, one administered vehicle and the other TEE. The animals were orally fed with a dose of 400 mg/kg using a feeding needle once daily for six weeks and were sacrificed six hours after the last administration. For vehicle-treated young and aged mice, the same volume (i.e., 0.2 mL) of 0.5% carboxymethyl cellulose-sodium solution was administered once daily for six weeks (Figure 1A). Each group composed of nine mice and vehicle-treated young mice were used as positive control.

### 2.4. Novel Object Recognition (NOR) Test

To investigate whether TEE has a cognition-enhancing effect, TEE was orally administered to 12-month-old aged mice for 6 weeks, and the NOR test, a non-forced recognition memory test, was performed using a modified method [19]. Briefly, the test apparatus consists of an opaque plastic chamber (25 cm × 25 cm × 25 cm), and the procedure includes three phases: habituation, training and testing. On the first day, the mice were allowed to freely explore the chamber without objects for 5 min to be familiarized with the environment (habituation phase). On the second day, the mice were given 5 min of exposure using an identical pair of objects (training phase). For short-term memory retention, the mice were placed in the chamber 1 h later with one of the previous objects and a novel object for 5 min in the testing phase (Figure 1B). Both training and testing sessions were recorded with a video camera and were analyzed by investigators blinded to the group allocation.

### 2.5. Sample Collection

The mice were anesthetized with an intramuscular injection of Zoletil (Virbac, Fort Worth, TX, USA) and xylazine solution (3:1 ratio) and were transcardially perfused with 0.9% normal saline. The brains were carefully dissected and were separated into two hemispheres. For the biochemical analyses, the hippocampus was removed from the left hemisphere, snap-frozen in liquid nitrogen, and stored at −80 °C. The right hemisphere was fixed with 4% paraformaldehyde in phosphate buffered saline (PBS) overnight at 4 °C and was equilibrated in 30% sucrose. The brain tissues were cut into sequential coronal sections (40 μm thick) with a cryostat (Leica, Nussloch, Germany) and were individually collected in 24-multiwell culture plates.

### 2.6. DCX Immunohistochemistry

Immunostaining for DCX was performed using the free-floating technique. One series was randomly selected and stained using antibodies against DCX (Santa Cruz Biotechnology, Dallas, TX, USA). The free-floating slices were incubated for two days at 4 °C with the primary antibodies in a diluent buffer comprising 1% bovine serum albumin (BSA)(Sigma-Aldrich, St. Louis, MO, USA) and 1% Triton X-100 in 0.1 M phosphate buffer. Subsequently, the sections were incubated for 24 h at 4 °C with biotinylated rabbit anti-goat IgG antibody (Vector Laboratories Ltd., Burlingame, CA, USA). The sections were then incubated with a Vector ABC kit (Vector Laboratories Ltd., Burlingame, CA, USA). DCX-positive cells were visualized with 3,3′-diaminobenzidine (DAB) (Vector Laboratories Ltd., Burlingame, CA, USA), and the images were taken with a Leica DM5500B microscope (Leica) (Leica Microsystems, GmbH, Wetzlar, Germany). To quantify the total number of DCX-positive cells in the SGZ, all sections were coded, and cell counting was performed with the examiner blinded to the group allocation.

### 2.7. Enzyme-Linked Immunosorbent Assay (ELISA)

The hippocampus was homogenized in lysis buffer (20 mM Tris, 137 mM NaCl, 1% NP-40 detergent, 10% glycerol, 1 mM phenylmethylsulfonylfluoride, (PMSF), 10 μg/mL aprotinin, 1 μg/mL leupeptin, and 0.5 mM sodium orthovanadate; pH 7.2) and then centrifuged at 15,000× *g* for 15 min at 4 °C, and the supernatants were used for analysis. ELISA was performed using the Corticosterone ELISA kit (Enzo Life Sciences, Farmingdale, NY, USA) to measure the endogenous corticosterone and the BDNF Emax Immunoassay System (Promega, Madison, WI, USA), to measure the BDNF levels in the hippocampus. All procedures were carried out according to the manufacturer’s instructions. The corticosterone and BDNF levels were quantified at 450 nm using an ELISA reader (Thermo Fisher Scientific Inc., Waltham, MA, USA) and were analyzed by a standard curve.

### 2.8. Western Blot Analysis for ERK and PSD95

A western blot analysis was performed as previously described [20]. The protein quantity was determined using a bicinchoninic acid (BCA) reagent (Sigma-Aldrich). Twenty grams of protein extract was separated by sodium dodecyl sulfate-polyacrylamide gel electrophoresis (SDS-PAGE) and were then transferred to the polyvinylidene difluoride (PVDF) membrane (Amersham, Buckinghamshire, UK). The membrane was blocked in 5% fat-free milk in Tris-buffered saline with Tween 20 (TBST) (20 mM Tris-HCl, pH 7.6, containing 0.4% Tween 20) and was incubated with primary antibodies for 24 h. The membrane was further incubated for 1 h with horseradish peroxidase-conjugated secondary antibody. The proteins were visualized using an enhanced chemiluminescence (ECL) detection system (GE Healthcare, Little Chalfont, UK).

### 2.9. Statistical Analyses

The behavioral data were analyzed using unpaired Student’s *t*-tests, and a *p*-value of less than 0.05 was considered to be statistically significant. The data from the immunohistochemistry, western blot and ELISA were analyzed using one-way ANOVA with Newman-Kuels post hoc tests for the multiple comparisons tests. The results are expressed as means ± standard error of the mean (SEM), and the statistical analyses were performed using the SPSS 22.0 software (IBM, Armonk, NY, USA).

## 3. Results

### 3.1. Oral TEE Supplement Did Not Affect Body Weight in Aged Mice

A total of eighteen mice (nine mice/group) completed the study. To examine whether TEE administration was associated with a change in the body weight of mice, we measured the body weight every week. Six weeks of TEE treatment did not influence the body weight of the mice when compared to the vehicle-treated group (Figure 1C).

### 3.2. Oral TEE Supplement Improved Age-Related Memory Impairment

**Figure 2:**
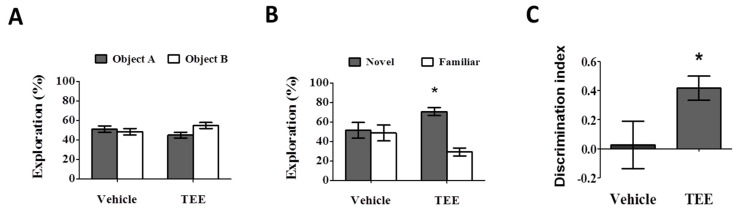
Oral administration of TEE improved age-related memory impairment. The novel object recognition test was performed to identify a preference to explore objects in the vehicle or TEE-treated mice (*n* = 9 per group). (**A**) Training phase. Data are presented as the percentage of exploration time to two identical objects; (**B**) Test phase. Data are presented as the percentage of exploration time to a novel or familiar object. (*, *p* < 0.05, versus familiar object); (**C**) The discrimination index was calculated as the difference between the exploring time to novel object (N) and familiar object (F), divided by the total time exploring both objects (discrimination index = (N − F)/(N + F). Asterisks denote a significant difference, *, *p* < 0.05, versus vehicle-treated group).

During the training phase, there were no statistical differences in the percentage of time spent exploring two identical objects (Figure 2A). During the test phase, the vehicle-treated mice also showed a comparable percentage of time spent exploring the novel object (51.4% ± 8.2%). However, the group with oral administration of TEE exhibited a significant increase in the percentage of time spent exploring the novel object, (70.8% ± 4.1%) compared to familiar object (29.2% ± 4.1%), as can be seen in Figure 2B. As shown in Figure 2C, the discrimination index in the TEE-treated mice (0.4 ± 0.1) was significantly higher than that of the vehicle-treated mice (0.0 ± 0.2). Thus, TEE administration could improve cognitive function in aged mice, suggesting that TEE might have a cognition-enhancing effect against age-related memory decline.

### 3.3. Oral TEE Supplement Increased DCX^+^ Cells and PSD95 Protein Expression in Aged Mice

**Figure 3:**
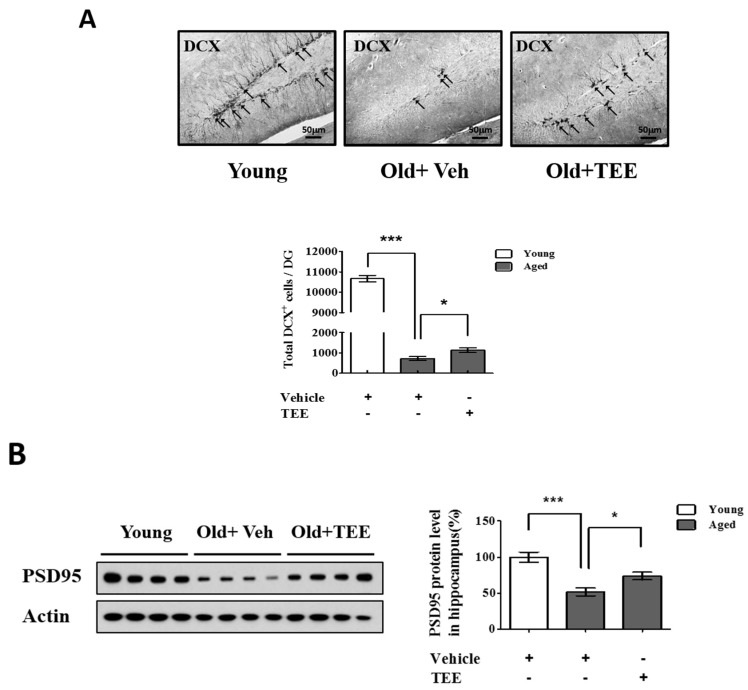
Oral administration of TEE improved hippocampal neurogenesis and synaptic density in aged mice. (**A**) To quantify the neurogenesis in dentate gyrus (DG), doublecortin (DCX) immunostaining was performed. Representative photographs of DCX^+^ cells in the hippocampal region are shown. The arrows indicate DCX^+^ cells, and the total number of DCX^+^ cells in the DG were quantified in the graph in the lower panel; (**B**) The expression of postsynaptic density-95 (PSD-95) was assessed via Western blotting. The bands shown are four representatives from each group. Relative protein expressions of PSD-95 were analyzed using the ImageJ software. The band intensity was normalized to actin. Each bar represents the mean ± SEM of each group (*n* = 9). The asterisks denote a significant difference (*, *p* < 0.05; ***, *p* < 0.001).

The above results indicated that the TEE treatment enhanced cognitive function in aged mice. To investigate whether the TEE treatment affected hippocampal neurogenesis, the overall cell proliferation in the SGZ of the dentate gyrus (DG) was assessed via immunohistochemistry for DCX, a marker for immature neurons. We found a significant reduction (14.8-fold) in the number of DCX^+^ cells in the DG in aged mice (12 months of age) compared to young (8-week-old) mice (720.0 ± 101.2 vs. 10667.5 ± 157.9; aged vs. young). However, the mice supplemented with TEE showed a significant increase (1.6-fold) in the number of DCX^+^ cells in DG (720.0 ± 101.2 vs. 1132.0 ± 117.9; vehicle vs. TEE), as shown in Figure 3A. In addition, we examined the PSD95 protein levels in the hippocampus because PSD95 is involved in synapse formation and synaptic plasticity. The age-related reduction of PSD95 expression in aged mice was significantly up-regulated in the TEE-supplemented group (Figure 3B). These findings suggest that the cognition-enhancing effect of TEE is a result of the induction of hippocampal neurogenesis and synaptic plasticity in aged mice.

### 3.4. Oral TEE Supplement Decreased Corticosterone and Increased BDNF in Hippocampus

**Figure 4:**
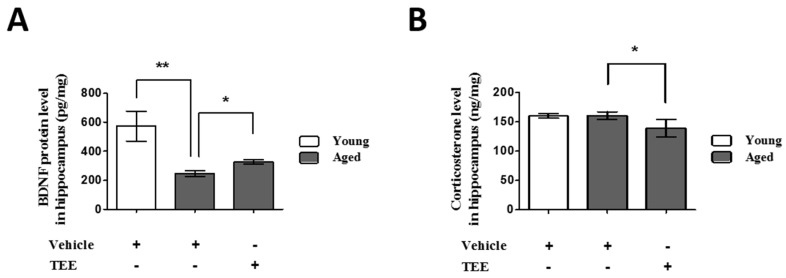
Oral administration of TEE decreased corticosterone and increased brain-derived neurotrophic factor (BDNF) in in aged hippocampus. The corticosterone (**A**) and BDNF (**B**) levels in the mouse hippocampus were measured using ELISA. Each bar represents the mean ± SEM for each group (*n* = 9). The asterisks denote a significant difference (*, *p* < 0.05; **, *p* < 0.01).

To investigate the possible mechanisms of cognition enhancement and neurogenesis for TEE, we analyzed the corticosterone and BDNF levels in hippocampus via ELISA. Although BDNF is significantly decreased in the hippocampus of aged mice when compared to young mice, TEE administration significantly increased the hippocampal BDNF protein by 32.6% ± 6.7% in aged mice (Figure 4A). There was no statistical difference in the hippocampal corticosterone levels between young and aged mice. However, the levels of corticosterone in the TEE-treated mice hippocampus decreased significantly by 13.3% ± 3.5% relative to vehicle-treated aged mice (Figure 4B).

### 3.5. Oral TEE Supplement Activated ERK/CREB Signaling Pathway

**Figure 5:**
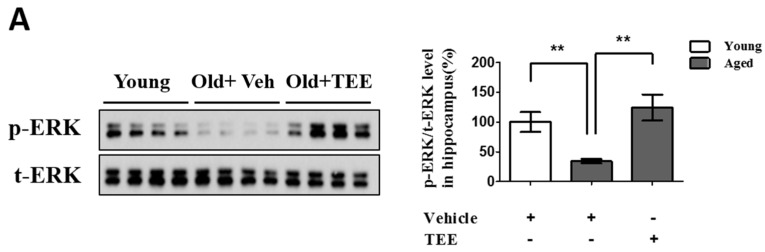

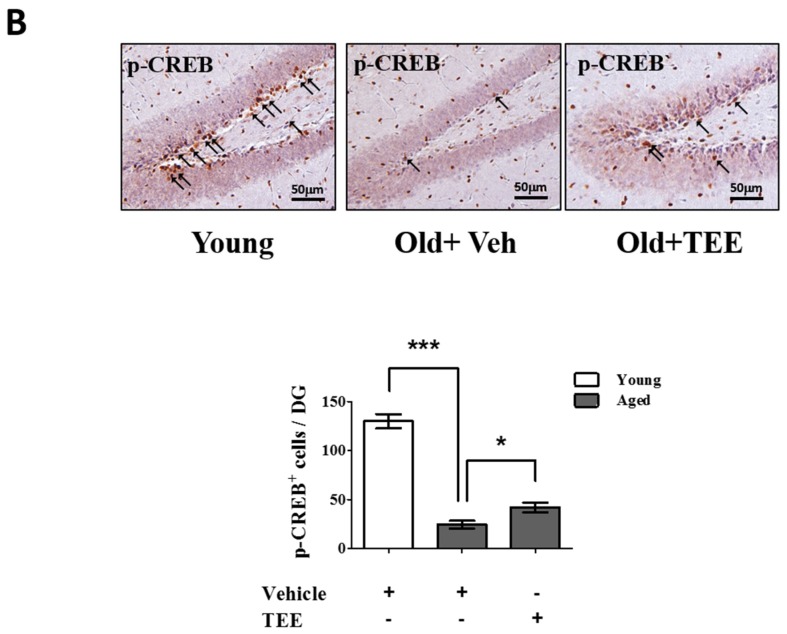
Oral administration of TEE activated extracellular-signal-regulated kinase (ERK) and cAMP response element binding (CREB) signaling pathways in aged hippocampus. (**A**) The changes in the phosphorylated form of ERK in the mice hippocampus were analyzed via Western blotting using phospho-specific ERK. The bands shown are four representatives from each group. The relative protein expressions of phospho-ERK were analyzed using the ImageJ software. The intensity of the bands was normalized to the total ERK; (**B**) To analyze the CREB activation in DG, phospho-CREB immunostaining was performed. Representative photographs of phospho-CREB^+^ cells in the hippocampal DG are shown. The arrows indicate phospho-CREB^+^ cells. The number of phospho-CREB^+^ cells in the DG was quantified in the graph in the lower panel. Each bar represents the mean ± SEM of each group (*n* = 9). The asterisks denote a significant difference (*, *p* < 0.05; **, *p* < 0.01; ***, *p* < 0.001).

Chronic exposure to corticosterone down-regulates the BDNF expression, and decreases neurogenesis in the hippocampus [21]. In addition, the activation of the ERK/CREB signaling pathway induced by BDNF plays a critical role in hippocampal neurogenesis and cognition improvement [22]. To examine whether the TEE supplement led to the activation of ERK and CREB in the hippocampus, we investigated the changes in the phosphorylation of ERK and CREB via Western blot analysis and immunohistochemistry, respectively. TEE administration significantly increased the levels of ERK phosphorylation in the hippocampus by 262.5% relative to vehicle-treated aged mice (Figure 5A). Furthermore, the TEE treatment significantly induced the phosphorylation of CREB in the SGZ of DG (Figure 5B). These results indicate that the TEE supplement can improve the age-dependent decrease in the hippocampal neurogenesis and cognition by activating the ERK/CREB pathway as well as increasing the BDNF production.

## 4. Discussion

The decline in adult neurogenesis and subsequent memory impairment is a major event associated with aging that is referred to as neurodegeneration [23], and the age-related decline in neurotrophic factors is considered to be one of the major causes of neurodegeneration [24]. Thus, continuous efforts have been made to supplement these factors or their analogs and to develop agents to restore their expression.

Over the past decade, natural ingredients from plants, vegetables, and fruits have been used as sources of pharmaceutical agents. For example, flavonoids from many kinds of plants have diverse pharmacological activities, including anti-oxidant, anti-inflammatory and anti-cancer effects [25,26], and the development of novel drugs from edible natural sources is of growing interest since fewer side effects are expected than when synthetic drugs are used. In this study, we examined effects of TEE on cognitive function in aged mice and elucidated the underlying mechanisms that are involved.

The cognitive function was evaluated using the NOR test, which is one of the most frequently used methods for assessing memory alterations in various subfields within neuroscience [27]. During the training session, all mice groups showed a similar preference for two identical objects. However, during the test session, the TEE-treated mice showed a significantly stronger interest in the novel object than the vehicle-treated mice did, indicating that the TEE supplement led to a cognitive enhancement in the aged mice.

To support our behavioral test, we investigated the morphological changes and proliferation of neuronal cells in the DG, as determined via DCX immunostaining. DCX plays a critical role in promoting microtubule polymerization, and it is expressed in neuronal precursor cells and immature neurons [28]. In general, DCX protein has been used as a neurogenesis marker that is exclusively expressed in developing neurons. In our present study, the DCX expression in the DG of aged mice decreased dramatically relative to that of young mice, which is consistent with previous reports [29]. However, these reductions were partially but significantly restored by oral supplementation of TEE for six weeks. In addition, TEE supplementation also increased the synaptogenesis or synaptic plasticity, as shown by an increased expression of PSD95 (a postsynaptic marker). These results suggest that the cognition-enhancing effects of TEE on aged mice were a result of the activation of neuronal cell proliferation in DG.

Oral TEE treatment significantly increased the age-related reduction in BDNF expression in the hippocampus. To confirm the effect of TEE, we investigated whether TEE enhances BDNF production in brain tissues isolated from young mice. ICR mice (female, six weeks) were divided two groups; vehicle-treated group and TEE-treated group. The mice were orally administered with 400 mg/kg of TEE every day for two weeks. As a result, the mRNA level of BDNF in TEE-treated group was significantly increased (1.89-fold) compared with vehicle-treated group (1.0 ± 0.37 vs. 1.89 ± 0.73; vehicle vs. TEE), as shown in Figure S1. Furthermore, we also investigated the effect of TEE on the production of BDNF and NGF in astrocytes. The treatment of TEE increased the protein level of BDNF in astrocytes, but not NGF as follows; (vehicle vs. TEE treatment, BDNF (pg/mL); 16 ± 0 vs. 70 ± 2). These results indicated that the administration of TEE could help to promote the development of neurogenesis and to protect the brain aging through enhancement of BDNF production.

Corticosterone levels in hippocampus were significantly decreased in the TEE-treated mice. Corticosterone is known as a stress hormone, and it is also associated with cognitive impairment [30]. Recent studies have demonstrated that increased levels of hippocampal corticosterone exhibit a poorer spatial memory performance [31], and chronic corticosterone exposure suppresses the synaptic plasticity of the mice brain [32]. Furthermore, corticosterone treatment decreased the BDNF expression in a mouse model as well as in a hippocampal cell line [33,34]. Our results indicate that the suppression of the hippocampal corticosterone levels by TEE is also involved in the BDNF induction and cognition improvement.

BDNF is a neurotrophic factor involved in neurogenesis, the modulation of synaptic plasticity, and the release of neurotransmitters [35]. BDNF-transgenic mice showed an improved cognitive function and synaptic plasticity [36] whereas BDNF knockout mice showed compromised learning and memory in spatial learning [37]. Several agents, inducing BDNF levels in the hippocampus, led to memory enhancement in mouse models [38,39]. Moreover, an oral supplementation of TEE significantly activates the phosphorylation of ERK and CREB in the murine hippocampus. ERK1/2 is a downstream target for the BDNF signaling pathway [40], and BDNF-induced ERK activation sequentially activates the transcription factor CREB protein and neurogenic proteins that are involved in memory formation and synaptic remodeling [41]. In addition, ERK signaling is closely related with the induction and maintenance of long-term potentiation [42]. Therefore, the activation of the ERK and CREB pathway in neurons is considered to be an effective therapeutic strategy for memory impairment and related disorders. Our results indicate that the BDNF-ERK-CREB pathway is involved in the cognition-enhancing effects of TEE.

Lycopene and rutin have been known as active compounds of tomato. To reveal active components in TEE, we analyzed components in TEE using HPLC and LC/MS. However, high concentrated peaks were not found, which are able to represent functionality of TEE. In the case of lycopene, it was included at 0.0001478% in TEE (data not shown). In addition, rutin was also included at 0.00696% in TEE. These result indicated that polyphenols, flavonoids, and non-polar components reported as functional components in tomatoes showed low possibilities of being active components in the functionality of TEE. Therefore, we suggest that the enhancing effect of TEE on cognitive function via BDNF production might be derived from poly/oligo-saccharides because TEE (50% ethanolic extract of tomato) contained various hydrophilic saccharides [43,44].

Animal models with cognitive impairment have been used as a tool to evaluate cognitive functions since these reflect the complex interactions among diverse neural systems. Several new mouse models are now available with cognitive impairment induced by pharmacologic, toxicological, and genetic methods [45]. Although these animal models have been successfully applied to investigate memory disorders, such as Alzheimer’s disease [46] and PD [47], the age-related cognitive decline is not fully explained using these artificially-induced models. Although a relatively longer time and additional resources are needed in order to use naturally-aged mice, we employed naturally-aged mice to recapitulate the aging systemic milieu [48] and subsequent response to TEE supplementation.

## 5. Conclusions

In this study, we showed that oral administration of TEE for 6 weeks enhanced cognition in aged mice. The TEE supplement improved the age-related reduction of hippocampal neurogenesis, increased BDNF production, and increased the levels of phosphorylated ERK and CREB in the hippocampus. Taken together, the cognition-enhancing effect of TEE might be attributed to increased neurogenesis and synapse formation in the hippocampus via activation of the BDNF signaling pathway. These results suggest that TEE can be a potential candidate for treating age-related memory impairment and neurodegenerative disorders. Furthermore, we suggest that increased consumption of tomato (salad or tomato-based food) would contribute to protect against brain aging.

## Supplementary Materials

The following are available online at http://www.mdpi.com/2072-6643/8/11/679/s1, Figure S1: Effect of TEE administration on brain-derived neurotrophic factor (BDNF) expression in the mouse brain.
