# Supplementary material for: Lycopersicon esculentum Extract Enhances Cognitive Function and Hippocampal Neurogenesis in Aged Mice
Source: Nutrients. 2016 Oct 26;8(11):679. doi: 10.3390/nu8110679 (PMC5133067; doi:10.3390/nu8110679)
Supplement: Supplementary file 1 [file nutrients-08-00679-s001.docx]

Supplementary Materials: *Lycopersicon esculentum* Extract Enhances Cognitive Function and Hippocampal Neurogenesis in Aged Mice

Jung-Soo Bae, Mira Han, Hee Soon Shin, Dong-Hwa Shon, Soon-Tae Lee, Chang-Yup Shin, Yuri Lee, Dong Hun Lee and Jin Ho Chung

**A**


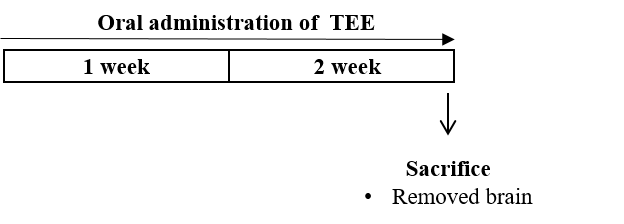


**B**


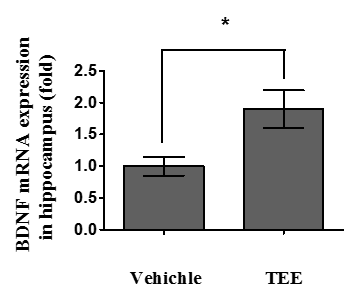


**Figure S1.** Effect of TEE administration on brain-derived neurotrophic factor (BDNF) expression in the mouse brain. (**A**) Schamatic design of the experiment of in vivo. Female 5 weeks ICR mice were divided into two groups: vehicle and TEE. Mice were orally provided with 400 mg/kg of TEE from days 0 to 21. On last experimental day, mice were sacrificed and removed brains for hormone analysis; (**B**) The brain was collected and homogenized. Homogenized brain was extracted mRNA and cDNA was synthesized using the QuantiTect Reverse Transcription Kit (Qiagen). Quantification of the BDNF mRNA level by quantitative real-time RT-PCR. Expression levels were normalized to Gapdh. The expression of BDNF expression was compared between the vehicle and TEE–treated groups. Each bar represents the mean ± SEM of each group (*n* = 6). The asterisks denote a significant difference (*, *p* < 0.05).
